# Underwater Weight Estimation of Three Sea Cucumber Species in Culture Tanks Using Image Analysis and ArUco Markers

**DOI:** 10.3390/ani15081121

**Published:** 2025-04-13

**Authors:** Roongparit Jongjaraunsuk, Saroj Rermdumri, Kanokwan Khaodon, Alongot Intarachart, Wara Taparhudee

**Affiliations:** 1 Department of Aquaculture, Faculty of Fisheries, Kasetsart University, Bangkok 10900, Thailand; ffisrrj@ku.ac.th; 2 Sriracha Fisheries Research Station, Center for Research and Academic Support Administration, Faculty of Fisheries, Kasetsart University, Bangkok 10900, Thailand; ffissrr@ku.ac.th (S.R.); ffiskwk@ku.ac.th (K.K.)

**Keywords:** underwater weight estimation, sea cucumber, image analysis, ArUco marker

## Abstract

Sea cucumbers play a vital role in marine and coastal ecosystems, with some species holding significant economic value. Accurate growth assessment, particularly weight estimation, is crucial for their management and conservation. However, direct measurement poses challenges, as sea cucumbers expel internal fluids when handled, altering their body size and weight. This study evaluates the effectiveness of image analysis combined with ArUco markers to estimate the weight of three economically and ecologically important sea cucumber species found in Thailand: black sea cucumber (*Holothuria leucospilota*), pink warty sea cucumber (*Cercodemas anceps*), and sandfish (*Holothuria scabra*). The proposed method demonstrated high accuracy, with R^2^ values of 0.9699, 0.9774, and 0.9882, respectively. Furthermore, no significant differences (*p* > 0.05) were observed between traditional hand measurements and image-based assessments, with relative errors of 7.71 ± 4.30% for black sea cucumber, 5.06 ± 3.37% for pink warty sea cucumber, and 4.50 ± 3.23% for sandfish. Unlike deep learning, which requires large datasets and high computation, this simple, cost-effective, and adaptable method highlights the potential of image analysis combined with ArUco markers as a non-invasive and accurate tool for estimating sea cucumber weight. This approach minimizes stress on the animals and can be extended to other aquatic species. However, challenges such as shadows, water turbidity, and the presence of similarly shaped objects near sea cucumbers should be considered when applying this technique in field conditions.

## Simple Summary

Sea cucumbers are ecologically important in marine ecosystems, and some species are valuable for trade. Studying their growth is important, but measuring them is difficult because they shrink and lose weight when touched. This study tested a new way to estimate their weight using photos and ArUco markers instead of handling them. The method was tested on three sea cucumber species found in Thailand: black sea cucumber, pink warty sea cucumber, and sand fish. The results showed that this technique is very accurate. This approach can help study their growth without causing stress and may also be useful for other animals. However, challenges include shadows, water clarity, and objects that look similar to sea cucumbers.

## 1. Introduction

Sea cucumbers have been valued in Asia for over 1000 years, and rising demand has led to overfishing, particularly in Asia, with traders seeking sea cucumbers from other regions. The most traded species belong to the order Aspidochirotida [1]. Global fishing now spans both small-scale tropical operations and industrial methods in temperate areas [2]. In 2022, 42,000 tonnes were captured globally, with Japan’s aquaculture contributing 256,000 tonnes [3]. Overfishing, lack of conservation, and habitat loss continue to threaten sea cucumber populations, with most of Asia’s supply coming from capture fisheries [4].

According to the Biodiversity-Based Economy Development Office (Public Organization) or BIDO [5], Thailand is home to four orders, nine families, and 123 sea cucumber species. Of these, 61 species are found along the Gulf of Thailand, 66 along the Andaman Sea, and 27 in both regions. The confirmed classifications include four orders, eight families, 28 genera, and 97 species.

This study focuses on three economically and ecologically significant species: black sea cucumber (*Holothuria leucospilota*), pink warty sea cucumber (*Cercodemas anceps*), and sandfish (*Holothuria scabra*). These species inhabit different ecosystems and have varying economic values. Regular population monitoring is particularly important for sandfish, which is harvested for consumption and export and is classified as endangered. It is one of Thailand’s most economically valuable sea cucumber species [6]. Fresh sandfish fetch between 9 and 15 US dollars per kilogram, while dried sandfish command higher prices, ranging from 80 to 200 US dollars per kilogram [5]. Given its economic significance, conservation efforts and aquaculture initiatives, including cultivation in earthen and concrete ponds, have gained increasing attention.

Growth studies typically assess body length and weight. Previous research has explored weight estimation using length measurements. For example, a study on sandfish farming in Ban Koh Pu, Nuea Khlong District, Krabi Province, found the relationship W = 5.1262 L^1.4992^ [6]. Similarly, Phattharapongphan et al. [7] reported a relationship of W = 0.0157 TL^1.7793^ for sandfish from Libong Island, Trang Province. Both studies observed allometric growth patterns with 95% confidence intervals. However, this approach requires handling the sea cucumbers, which induces stress and causes water loss, leading to underestimation of true weight.

Advancements in image processing, machine learning, and deep learning have enabled non-invasive methods for size and weight assessment in aquatic animals. Such techniques minimize handling, reducing stress and injury risks [8]. For instance, Petrellis [9] integrated image processing and machine learning for non-invasive fish morphological monitoring, achieving length estimation errors between 1.9% and 13.2%. Nguyen et al. [10] applied the AquaAttSeg model, a deep-learning-based segmentation technique, to improve fish size measurement accuracy in natural habitats. Similarly, Chirdchoo et al. [11] used image processing to detect individual shrimp and extract morphological features such as area and length.

Several studies have applied image analysis to sea cucumbers. Lee et al. [12] used a vision system to measure sea cucumber dimensions, achieving an R^2^ value of 0.999 for weight estimation through regression analysis. Qiao et al. [13] improved underwater image quality using histogram equalization and wavelet transformation techniques. Guo et al. [14] employed deep residual networks for underwater sea cucumber identification, while Guo et al. [15] developed a multi-scale enhancement fusion method based on human visual system modeling. Zhang et al. [16] proposed a deep-learning-based sea cucumber detection algorithm, and Zhu et al. [17] combined the YOLOv7 model with the GrabCut-RGBD method to achieve 97% accuracy in sea cucumber identification and precise size measurement.

However, these techniques often require large datasets with images captured in diverse environments to ensure high accuracy. Challenges such as variations in camera-to-subject distance can affect measurement reliability [18]. Additionally, in concrete or plastic pond aquaculture, only top-view images can be captured, limiting accuracy.

To address these limitations, ArUco markers have been introduced as reference tools for size adjustments in images. These markers help correct for distance variations, improving weight prediction reliability. They have been successfully applied in fish studies, such as weight estimation of Alaskan pollock through image analysis with reference markers [19]. When combined with machine vision systems, ArUco markers provide a non-destructive and efficient approach for size measurement [20] and enable real-time size estimation in aquaculture [21].

In this study, image processing and analysis techniques were integrated with ArUco markers to estimate the weight of three sea cucumber species, even with a limited image dataset. This approach aims to provide a proof-of-concept for further research in marine biology, ecology, and aquaculture.

## 2. Materials and Methods

### 2.1. Sampling and Nursery

Sea cucumbers were collected by scuba diving. Black sea cucumbers and pink warty sea cucumbers were obtained from Ao Sriracha, Sriracha District, Chonburi Province, while sandfish were collected from Koh Pu, Tambon Sri Boya, Amphoe Nuea Khlong, Krabi Province, Thailand. Approximately 20–50 individuals of each species were collected and transported to the Sriracha Fisheries Research Station, Faculty of Fisheries, Kasetsart University (101 12 Moo 9, Bang Phra Subdistrict, Sriracha District, Chonburi Province, Thailand).

The three sea cucumber species were housed separately in 2 × 2 m cement tanks, with seawater salinity maintained between 30 and 33 ppt and a water depth of 50–70 cm. Each tank was continuously aerated using five air stones throughout the rearing period. The tank floors were left bare, without added substrate (soil or sand). A flow-through seawater system was used instead of water exchange, allowing seawater to flow into the tanks for 3–6 h per day. No additional feed was provided, as the sea cucumbers obtained nutrients from plankton and organic matter in the seawater.

During the rearing period, water quality was measured daily in the morning (7:00–8:00 a.m.) to ensure optimal conditions. Dissolved oxygen (DO), water temperature (Temp), and pH were measured using a Rinko Profiler Multi-Parameter CTD Model ASTD 102 instrument (JFE Advantech Co., Ltd., Tokyo, Japan). Alkalinity (ALK), total ammonia nitrogen (TAN), and nitrite-nitrogen (NO_2_-N) were collected and analyzed in the laboratory following APHA [22] guidelines. The average water quality parameters recorded during the experiment were as follows: DO ranged from 7.95 to 8.23 mg/L, Temp varied between 28 and 30 °C, pH ranged from 7.90 to 8.15, ALK was between 108 and 125 mg/L, TAN ranged from 0.08 to 0.48 mg/L, and NO_2_-N was between 0.01 and 0.06 mg/L. These values fall within the optimal range for sea cucumber rearing [23,24,25].

### 2.2. Photography

**Figure 1:**
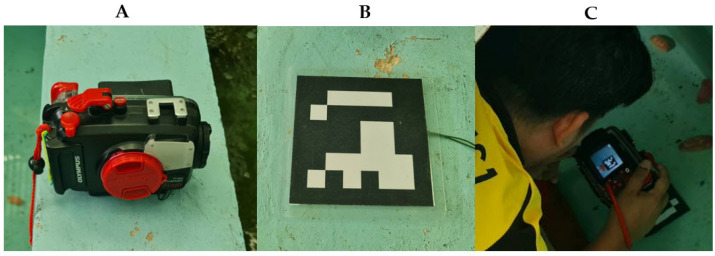
Underwater photography setup for sea cucumber image analysis using ArUco markers. (**A**) Olympus Tough PT-059 digital underwater camera, (**B**) ArUco marker sheet (6 × 6_250, Marker ID 1; 10 cm per side) coated with waterproof plastic, serving as a scale reference, and (**C**) sea cucumber sample (top view) positioned underwater alongside the marker, with a clean background to minimize image-analysis errors.

**Figure 2:**
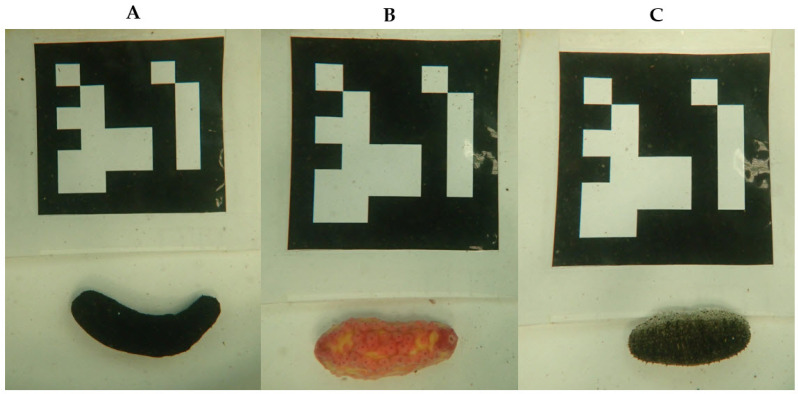
Sea cucumbers with ArUco markers—(**A**) black sea cucumber, (**B**) pink warty sea cucumber, and (**C**) sandfish.

Fifteen individuals of each sea cucumber species were sampled and photographed. In each image, an ArUco marker sheet (6 × 6_250, Marker ID 1), measuring 10 cm per side and coated with plastic for water protection, was placed next to the sea cucumber sample (Figure 1 and Figure 2). We used ArUco marker 6 × 6_250, Marker ID 1, to ensure accurate size and weight estimation of sea cucumbers underwater. This marker contains more bits than 4 × 4 or 5 × 5 markers, reducing the chances of misdetection and improving resistance to noise and distortions, thus providing high detection accuracy and consistency for correcting underwater distortions and standardizing measurements. The same marker pattern is used for all three species to maintain consistency across measurements, as the primary goal is to accurately assess the size and weight of the sea cucumbers rather than to distinguish between species. Photographs were taken using an Olympus Tough PT-059 digital underwater camera (Olympus Corporation, Tokyo, Japan) (Figure 1A). Imaging was conducted on a single day per tank (one day per species) in an area free from objects or stains to minimize potential errors in image analysis.

### 2.3. Weighing

**Figure 3:**
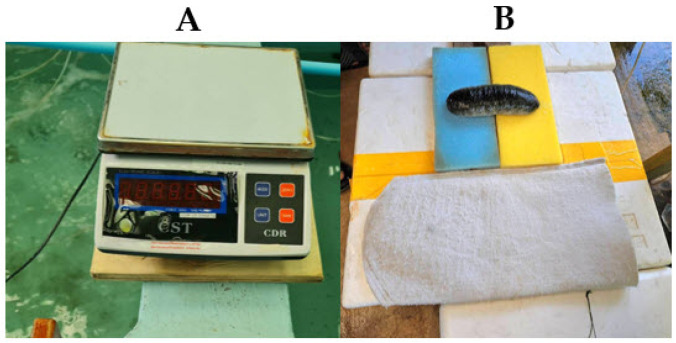
Traditional weight measurement procedure for sea cucumbers. (**A**) Digital CST-CDR scale (CST Instruments (Thailand) Ltd., Bangkok, Thailand) recording the actual weight, and (**B**) sea cucumber resting on a seawater-soaked cloth for 3–5 min to allow natural dehydration before weighing.

After underwater photography, each sea cucumber was gently placed on a seawater-soaked cloth for 3–5 min to allow for natural dehydration before weighing. This method ensured the most accurate weight measurement with minimal error, as it accounted for the natural water loss of the sea cucumber. Weighing was performed using a digital CST-CDR scale (CST Instruments (Thailand) Ltd., Bangkok, Thailand) as shown in Figure 3. The recorded weight data were compared with results obtained from the image-based analysis.

### 2.4. Image Analysis

**Figure 4:**
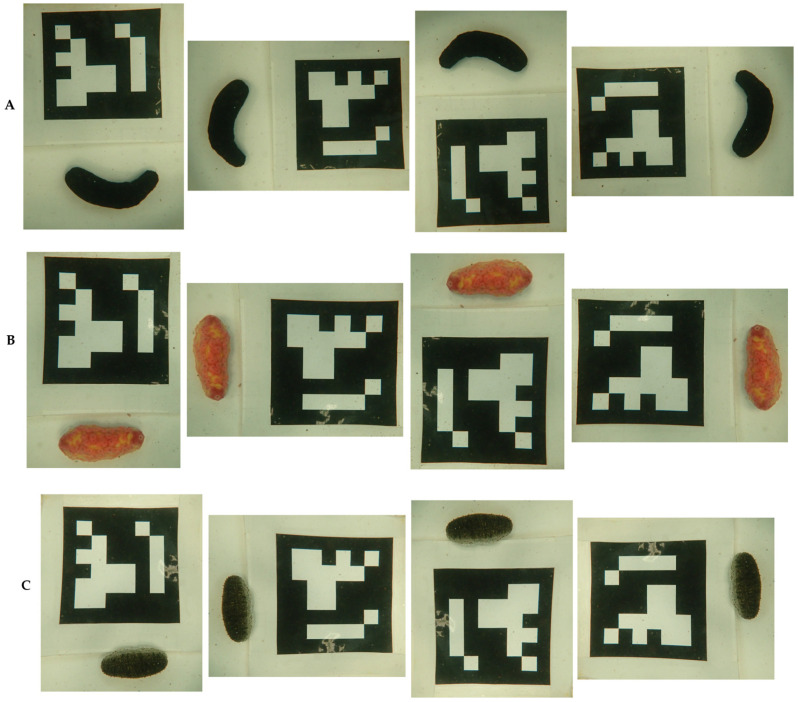
Augmented images of sea cucumber species for image analysis with (**A**) black sea cucumber, (**B**) pink warty sea cucumber, and (**C**) sandfish.

Before analysis, due to the limited number of image samples, images of all sea cucumber species were augmented by rotating them 90° clockwise (Figure 4), resulting in a total of 60 images per species. All images were then organized into separate folders named Black Sea Cucumber, Pink Warty Sea Cucumber, and Sandfish. These folders were uploaded to Google Drive and integrated into Google Colab for further processing.

The image analysis workflow focused on contour detection to analyze sea cucumber size and estimate weight, incorporating background removal with rembg, format conversion (RGBA to RGB and BGR for OpenCV), ArUco marker detection, Otsu’s thresholding for binarization, contour detection, area filtering, and object size calculation using ArUco markers. Valid contours were then drawn with bounding boxes and object details for analysis. The estimated weight was later compared with the actual weight for validation, aligning with established image processing frameworks and validation approaches [8,12,26]. A detailed breakdown of the workflow is provided in Table 1.

### 2.5. Sea Cucumber Weight Estimation

**Figure 5:**
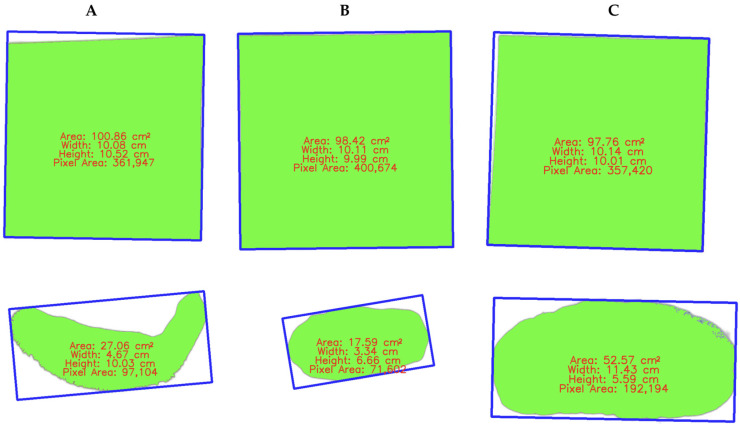
Image analysis results for sea cucumber weight estimation. The top row shows reference ArUco marker used for scale calibration. The bottom row displays processed binary images of sea cucumber with annotated measurements. The green-filled area represents the segmented region of the sea cucumber body; the blue rectangle indicates the bounding box used to calculate the object’s dimensions (width and height); and the red text overlay presents calculated area, width, height, and pixel area values with (**A**) black sea cucumbers, (**B**) pink warty sea cucumber, and (**C**) sandfish.

Each processed image was analyzed to determine the top-view area of the sea cucumber in square centimeters (Figure 5).

The relationship between the top-view area (x, cm^2^) and actual weight (y, g) was modeled using linear, polynomial, power, logarithmic, and exponential equations. Each model was applied to all species, and the equation with the highest coefficient of determination (R^2^) was selected for each species. The equations were generated using Python version 3.11.11 in the Google Colaboratory cloud–based environment.

### 2.6. Statistical Analysis

The best-fitting equation for each sea cucumber species was validated by comparing estimated weights with actual weights obtained via traditional methods. Before performing the independent *t*-test, we verified the assumptions of normality and homogeneity of variance. Normality was assessed using the Shapiro–Wilk test, and homogeneity of variance was checked using Levene’s test. An independent sample *t*-test was performed at a significance level of *p* < 0.05 using IBM SPSS Statistics version 26 (IBM Corp., Armonk, NY, USA).

### 2.7. Ethical Statement

All procedures adhered to applicable regulations and were conducted with approval from the Kasetsart University Institutional Animal Care and Use Committee (ACKU67–FIS–025), Bangkok, Thailand.

## 3. Results

### 3.1. Black Sea Cucumber

**Figure 6:**
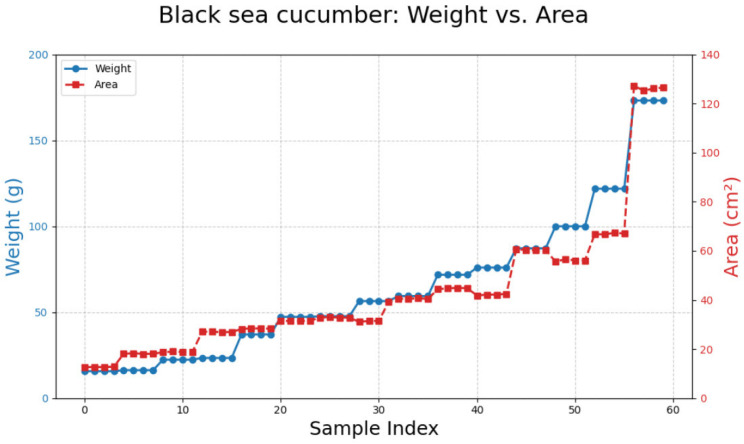
A comparison between weight and area measurements for black sea cucumbers.

**Figure 7:**
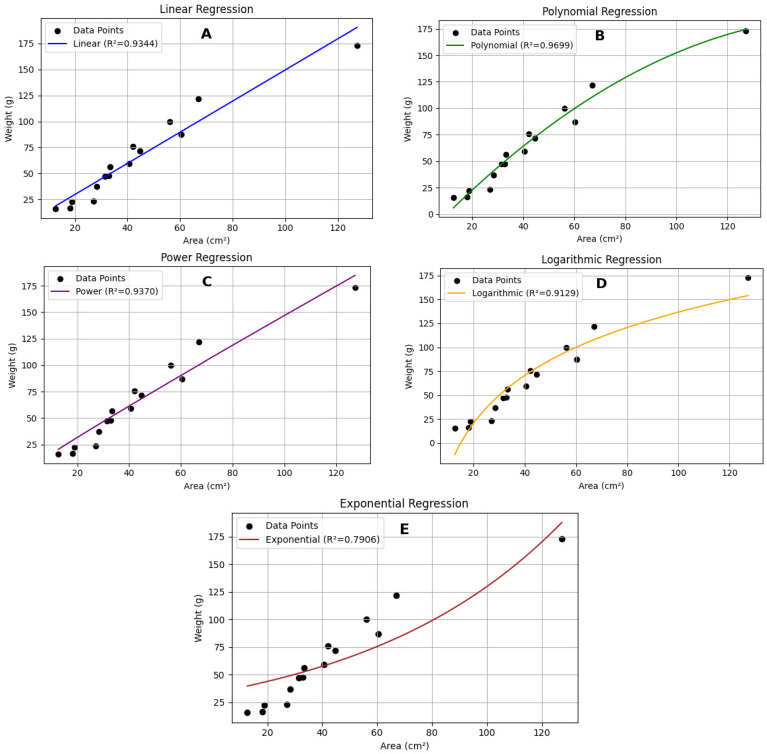
The relationship between weight (g) and area (cm^2^) of black sea cucumbers modeled using (**A**) linear, (**B**) polynomial, (**C**) power, (**D**) logarithmic, and (**E**) exponential trendlines.

Processing 60 images of black sea cucumbers (Figure 6) resulted in a polynomial equation: y = −0.0075x^2^ + 2.5255x − 24.8202. This equation yielded the highest coefficient of determination (R^2^ = 0.9699) compared to other models (Table 2; Figure 7).

### 3.2. Pink Warty Sea Cucumber

**Figure 8:**
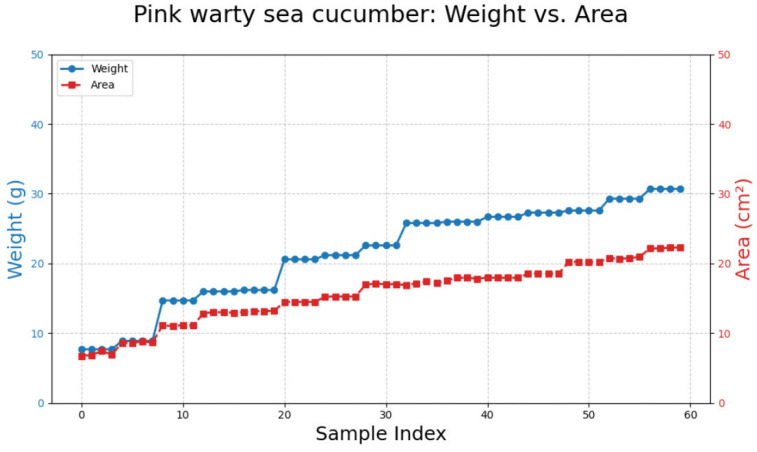
A comparison between weight and area measurements for pink warty sea cucumbers.

**Figure 9:**
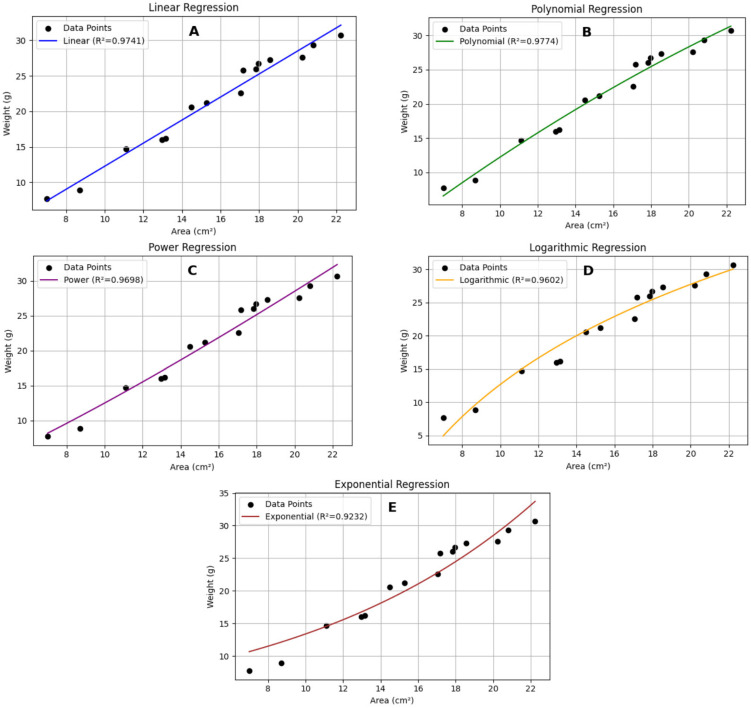
The relationship between weight (g) and area (cm^2^) of pink warty sea cucumber modeled using (**A**) linear, (**B**) polynomial, (**C**) power, (**D**) logarithmic, and (**E**) exponential trendlines.

Processing 60 images of pink warty sea cucumbers (Figure 8) resulted in a polynomial equation: y = −0.0209x^2^ + 2.2369x − 8.0638. This equation achieved the highest R^2^ value (0.9774) compared to other models (Table 3; Figure 9).

### 3.3. Sandfish

**Figure 10:**
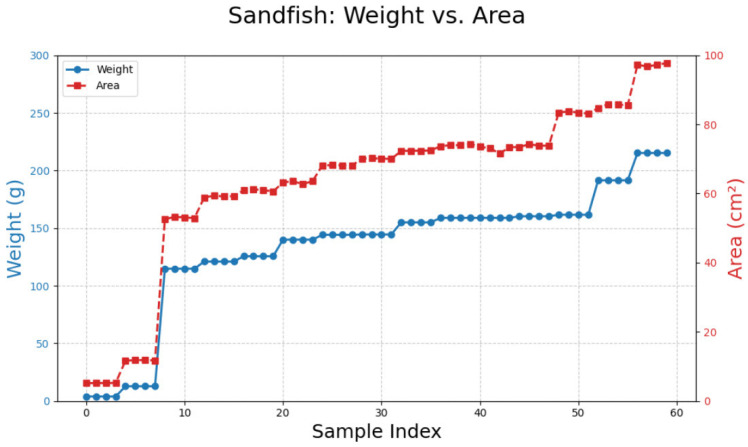
A comparison between weight and area measurements for sandfish.

**Figure 11:**
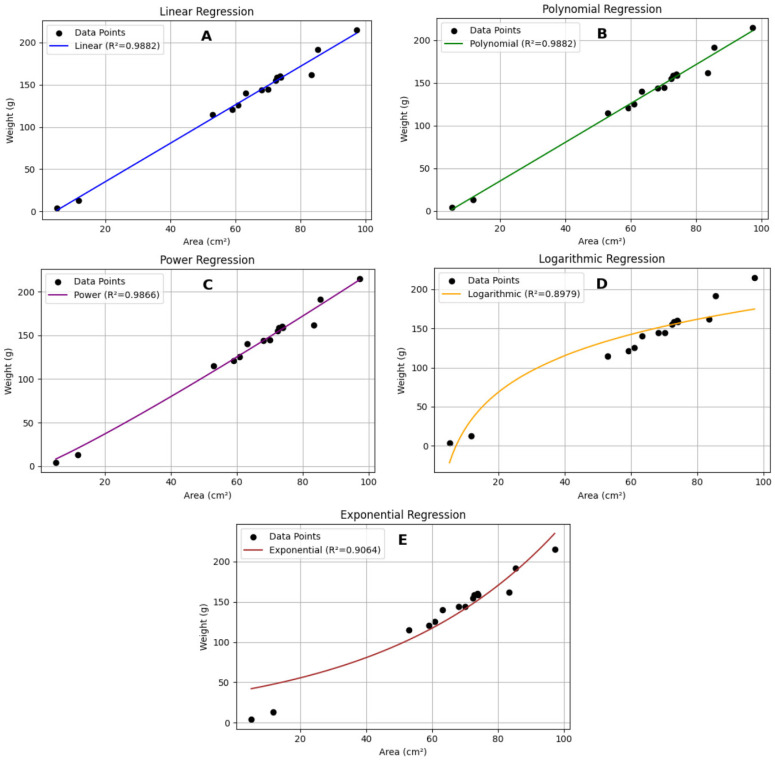
The relationship between weight (g) and area (cm^2^) of sandfish modeled using (**A**) linear, (**B**) polynomial, (**C**) power, (**D**) logarithmic, and (**E**) exponential trendlines.

Processing 60 images of sandfish (Figure 10) resulted in two equations with the highest R^2^ value (0.9882): y = 2.2761x − 10.3947 and y = 0.0002x^2^ + 2.2602x − 10.1545. These equations outperformed other models (Table 4; Figure 11).

### 3.4. Comparison of Results with the Conventional Method

**Figure 12:**
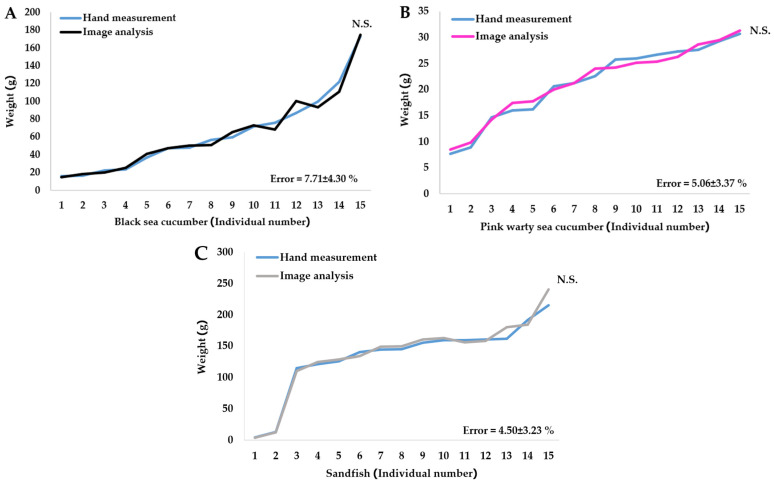
Comparison of weight estimation between hand measurement and image analysis for (**A**) black sea cucumber, (**B**) pink warty sea cucumber, and (**C**) sandfish. N.S.: no significant difference (*p* > 0.05).

The self-test results showed no statistically significant differences (*p* > 0.05) between the weight estimates obtained using image analysis and those obtained via traditional hand-weighing for all three species. The average error rates were 7.71 ± 4.30% for black sea cucumber, 5.06 ± 3.37% for pink warty sea cucumber, and 4.50 ± 3.23% for sandfish (Figure 12).

## 4. Discussion

The findings of this study highlight that image analysis techniques combined with ArUco markers can effectively estimate the weight of sea cucumbers from underwater photographs by establishing a relationship between body area and body weight. This approach achieved high accuracy, with R^2^ values of 0.9699 for black sea cucumbers, 0.9774 for pink warty sea cucumbers, and 0.9882 for sandfish. The accuracy of this method is comparable to previous studies that utilized various techniques for sea cucumber weight estimation, including those conducted by [12,13,14,15,16,17].

Some errors in the results may have arisen from lighting conditions, which can distort the perceived body area. Cesar et al. [27] noted that ArUco markers still face challenges due to environmental factors such as turbidity, lighting variations, and marker size, all of which can impact detection performance.

This study demonstrates that the proposed technique achieves accuracy comparable to other methods, including deep learning-based approaches. It offers several advantages, such as the effectiveness of ArUco markers in providing a reference scale within the image, enabling precise and consistent size measurements in real-world units. Accurate dimension or volume estimation is crucial for reliable weight assessment. Additionally, this technique requires less data, lower computational power, and shorter training times than deep learning models, which demand large datasets and significant resources for training. However, a direct comparison of operational costs and data requirements would further substantiate this advantage.

The limitation of this study is the small sample size, which hinders the construction of complex relationship equations. While we used 90° image rotation to increase the dataset size, this method may lack true variability in shape and perspective, potentially limiting generalization and causing overfitting. To mitigate this, future studies should explore additional augmentation techniques, such as random rotations, scaling, and slight perspective distortions, to enhance dataset diversity and reduce bias.

Furthermore, integrating advanced regularization techniques, such as dropout and L1/L2 weight regularization, can help prevent overfitting in deep learning-based models. Employing cross-validation, transfer learning, and early stopping will further ensure that the model generalizes well to unseen data.

Future research could also explore the use of different ArUco marker sizes combined with advanced machine vision and deep learning techniques to improve aquatic animal size estimation. By optimizing marker configurations and incorporating machine learning models, more precise and efficient methods can be developed to enhance monitoring and management in aquaculture and marine research.

Additionally, the integration of automatic segmentation techniques [13], Contrast Limited Adaptive Histogram Equalization (CLAHE), wavelet transformation, defogging enhancement methods [28], and the Retinex algorithm [29], along with a multi-scale convolutional hybrid attention residual network (MCRNet) [30], could significantly improve underwater image quality. These enhancements can lead to clearer images, reducing noise and distortion, thereby improving accuracy while minimizing overfitting risks in challenging aquatic environments.

## 5. Conclusions

This study demonstrates that image processing techniques combined with ArUco markers can effectively estimate the weight of sea cucumbers with high accuracy. The method was successfully applied to three economically and ecologically important species—black sea cucumber, pink warty sea cucumber, and sandfish—yielding R^2^ values of 0.9699, 0.9774, and 0.9882, respectively. This approach provides a non-invasive alternative to traditional weight measurement, preventing stress-induced fluid loss in sea cucumbers and ensuring more reliable growth assessments. With further refinement, this technique could be adapted for weight estimation in other aquatic organisms, contributing to improved monitoring and management of marine resources.

## Figures and Tables

**Table 1:** Image processing and object measurement workflow.

Process	Details
1. Install packages	Install rembg for background removal and onnxruntime for inference
2. Mount Google Drive	Access images from Google Drive
3. Import libraries	Load rembg, PIL, cv2, and numpy for processing
4. Set image paths	Define input and output paths
5. Open image	Read the image into memory
6. Remove background	Use rembg to remove the background
7. Apply transparency and white background	Convert to RGBA and place on a white background
8. Convert to RGB and save	Remove transparency and save the final image
9. Convert for OpenCV	Change to BGR format for processing
10. ArUco marker detection	Detect markers after grayscale conversion
11. Thresholding and contours	Apply Otsu’s thresholding and detect object contours
12. Filter and process contours	Set a minimum area (5 cm^2^), calculate object area, convert pixels to cm^2^ using ArUco markers, and analyze valid contours
13. Draw and overlay information	Display contours, bounding boxes, and object details
14. Show final image	Present the processed image with annotations

**Table 2:** Equations and coefficients of determination (R^2^) for different models of black sea cucumbers.

Model	Equation	R^2^
Linear	y = 1.4993x − 0.3209	0.9344
Polynomial	y = −0.0075x^2^ + 2.5255x − 24.8202	0.9699
Power	y = 1.8313x^0.9522^	0.9370
Logarithmic	y = −195.3286 + 72.1146 In(x)	0.9129
Exponential	y = 33.5828 × 10^0.0135x^	0.7906

**Table 3:** Equations and coefficients of determination (R^2^) of each study model of pink warty sea cucumbers.

Model	Equation	R^2^
Linear	y = 1.6247x − 3.9756	0.9741
Polynomial	y = −0.0209x^2^ + 2.2369x − 8.0638	0.9774
Power	y = 0.8102x^1.1886^	0.9698
Logarithmic	y = −37.3202 + 21.7212 In(x)	0.9602
Exponential	y = 6.2870 × 10^0.0756x^	0.9232

**Table 4:** Equations and coefficients of determination (R^2^) of each study model of sandfish.

Model	Equation	R^2^
Linear	y = 2.2761x − 10.3947	0.9882
Polynomial	y = 0.0002x^2^ + 2.2602x − 10.1545	0.9882
Power	y = 1.3355x^1.1086^	0.9866
Logarithmic	y = −132.1002 + 67.0347 In(x)	0.8979
Exponential	y = 38.1773 × 10^0.0187x^	0.9064

## Data Availability

The data supporting the findings of this study are available from the first and corresponding author upon reasonable request.
